# High-Performance Protonic Ceramic Fuel Cell with Ytterbium-Doped Barium Zirconate: Reducing Cathode Polarization by Improving Electrolyte Surface Condition

**DOI:** 10.1021/acsami.5c04806

**Published:** 2025-07-02

**Authors:** Hiroyuki Shimada, Konosuke Watanabe, Masaya Fujioka, Katsuhiro Nomura, Aman Sharma, Yuki Yamaguchi, Hirofumi Sumi, Yasunobu Mizutani

**Affiliations:** Innovative Functional Materials Research Institute, Department of Materials and Chemistry, 13508 National Institute of Advanced Industrial Science and Technology (AIST), 4-205 Sakurazaka, Moriyama-ku, Nagoya, Aichi 463-8560, Japan

**Keywords:** protonic ceramic fuel cell (PCFC), electrolyte surface, cathode polarization, power density, barium zirconate (BaZrO_3_)

## Abstract

Protonic ceramic fuel cells (PCFCs) have great potential to realize ultrahigh energy-conversion efficiency, but higher power density is required for future commercialization. The present work reports the effect of the electrolyte surface condition related to chemical composition such as stoichiometry and element distribution on cathode performance as a key for high-performance PCFCs. In our PCFCs, Ce-free Yb-doped BaZrO_3_ (BZYb20) electrolytes are prepared using two BZYb20 raw powder materials with different A/B ratios, i.e., Ba_0.97_Zr_0.8_Yb_0.2_O_3−δ_ (Cell-97) and Ba_0.99_Zr_0.8_Yb_0.2_O_3−δ_ (Cell-99). These PCFCs exhibit different element distributions on their BZYb20 electrolyte surfaces, namely, a heterogeneous element distribution with segregation of Yb_2_O_3_ for Cell-97 and a homogeneous distribution for Cell-99. The maximum power density of Cell-99 is higher than that of Cell-97 and reaches exceptionally high values, e.g., ∼1.3 W cm^–2^ at 600 °C and ∼0.7 W cm^–2^ at 500 °C, which are the highest attained for PCFCs with Ce-free BaZrO_3_-based electrolytes. The distribution of relaxation times and fitting analysis reveals that the cathode polarization resistance of Cell-99 is much lower than that of Cell-97 even when using the same cathode material. In conclusion, an optimal electrolyte surface condition significantly reduces cathode polarization resistance, leading to the achievement of high-performance PCFCs.

## Graphical abstract

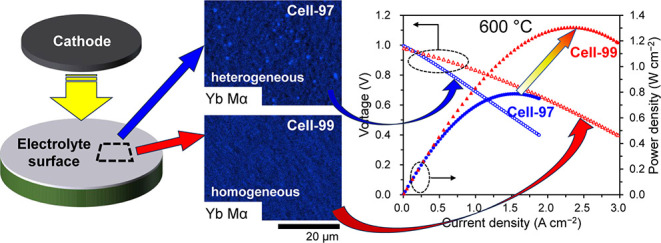

## 1 Introduction

High-temperature solid oxide fuel cells (SOFCs) currently have been introduced in the global market as a highly efficient power source that can be used in various scale applications, such as distributed cogeneration systems and monogeneration power plants.^1–4^ In addition, innovative novel electrolyte materials that can operate at lower temperatures have been developed to accelerate the widespread use of SOFCs.^5,6^ Recently, protonic ceramic fuel cells (PCFCs), which are proton-conducting-type SOFCs, have attracted much attention as a next-generation fuel cell.^7–10^ Although H_2_O is electrochemically produced at the anode side during operation in traditional oxide-ion-conducting SOFCs, H_2_O is produced at the cathode side in PCFCs. As a result, the introduced fuel is not diluted by H_2_O, and thus, PCFCs can operate at higher fuel utilization ratios than SOFCs.^11–16^ In addition, because the activation energy for proton conduction is lower than that of oxide-ion conduction, the operating temperature of PCFCs (400–700 °C) is lower than that of SOFCs (∼800 °C).^17^ Besides cost reduction, lowering the operating temperature contributes to an increase in the electromotive force of PCFCs. These features enable PCFCs to achieve higher energy-conversion efficiency (>70%, lower heating value, LHV) than SOFCs.^12,14–16^

Representative proton-conducting oxides are perovskite-type oxides, such as BaZrO_3_-based oxides (barium zirconates) and BaCeO_3_-based oxides (barium cerates). These oxides have relatively contrasting properties, namely, BaZrO_3_-based oxides have higher chemical stability against CO_2_ and lower proton conductivity and BaCeO_3_-based oxides have lower chemical stability and higher proton conductivity. The proton conductivity can be increased by doping trivalent cations such as Y^3+^ and Yb^3+^ into the B-sites of the perovskite oxides.^18–21^ In addition, Ba­(Zr,Ce)­O_3_-based oxides, in which Zr and Ce are simultaneously coplaced at the B-site, have been investigated to compatibly achieve both high proton conductivity and high chemical stability.^22–25^

Our research group has investigated PCFCs using Yb-doped BaZrO_3_, i.e., BaZr_0.8_Yb_0.2_O_3−δ_ (BZYb20), as the electrolyte material because BZYb20 has inherently high CO_2_ tolerance, relatively high proton conductivity among BaZrO_3_-based oxides, and good compatibility with Ni-containing anodes.^26,27^ Recent works have reported that PCFCs with the BZYb20 electrolyte have the potential for high energy-conversion efficiency and high durability. For example, we have developed a numerical model that can reproduce experimental data of PCFCs, and by using this model, we have shown the possibility of high energy-conversion efficiency exceeding 70% (LHV) of PCFCs with the BZYb20 electrolyte.^16^ In terms of durability, PCFCs with the BZYb20 electrolyte have demonstrated high long-term durability in 1000 h of operation at 600 °C; the degradation rate (cell voltage decrease rate) was 4.8% kh^–1^ and was further improved by 0.9% kh^–1^ in the case with a buffer interlayer of BaZr_0.1_Ce_0.7_Y_0.1_Yb_0.1_O_3−δ_ (BZCYYb1711) between the cathode and electrolyte.^28^

Although these superior properties make BZYb20 a candidate electrolyte material for PCFCs, higher power density, i.e., over 1 W cm^–2^ at intermediate temperatures of around 600 °C, is required to put the PCFCs into practical use. An important issue in PCFCs is the electrolyte surface condition related to chemical composition such as stoichiometry and element distribution due to Ba evaporation during the high-temperature sintering process. The decrease in Ba from the A-site of a BaZrO_3_-based oxide causes a decrease in both its proton conductivity and proton transport number.^29–32^ Although the decrease in Ba is only a few micrometers from the top surface of the electrolyte and might have a marginal effect on the overall proton conductivity of the electrolyte membrane, such a decrease can significantly affect the cathode performance. For example, Choi et al.^33^ reported the fabrication of a stoichiometric BaZr_0.4_Ce_0.4_Y_0.1_Yb_0.1_O_3−δ_ (BZCYYb4411) electrolyte by putting a BZCYYb4411 pellet as a Ba source onto the electrolyte during the sintering process, resulting in a PCFC with exceptional high power densities due to lowered ohmic and electrode polarization resistances. Bian et al.^34^ showed that an acid etching treatment effectively rejuvenated the BZCYYb1711 electrolyte surface in PCFCs, resulting in reactive bonding between the cathode and electrolyte and improved power density and stability.

In the present work, we investigate the effect of the surface condition of the BZYb20 electrolyte on the cathode performance by comparing PCFCs fabricated using two BZYb20 powder materials with different A-site to B-site ratios (A/B ratios), namely, an A/B ratio of 0.97 (Ba_0.97_Zr_0.8_Yb_0.2_O_3−δ_) and 0.99 (Ba_0.99_Zr_0.8_Yb_0.2_O_3−δ_). Note that to prevent the formation of BaCO_3_, we use BZYb20 powder materials, which are slightly deficient in Ba of the A-site cation. In addition, we demonstrate remarkable high power densities of the resulting PCFC with the BZYb20 electrolyte, which are the highest values in PCFCs with chemically stable Ce-free BaZrO_3_-based electrolytes.

## 2 Results and Discussion

### 2.1 Characteristics of BZYb20 Electrolytes on PCFCs

In the present work, anode-supported PCFCs were fabricated using two BZYb20 powders with different A/B ratios, i.e., Ba_0.97_Zr_0.8_Yb_0.2_O_3−δ_ and Ba_0.99_Zr_0.8_Yb_0.2_O_3−δ_, which are denoted here as Cell-97 and Cell-99, respectively (Table 1). Both PCFCs have an anode-supported configuration with a thin electrolyte layer (Figure S1) and have La_0.6_Ba_0.4_CoO_3−δ_ (LBC) and BaZr_0.9_Yb_0.1_O_3−δ_ (BZYb10) composite cathodes.^16^ For the electrolytes and anodes, we aimed to construct a microstructure that would be similar for both PCFCs by adjusting the cosintering temperature because the sinterability was different between these two BZYb20 materials; a higher A/B ratio generally leads to higher sinterability of BaZrO_3_-based oxides.^29–32^

**Table 1:** Specifications of Anode-Supported PCFCs Fabricated Using BZYb20 Electrolyte Powder Materials with Different A/B Ratios

	Electrolyte			Anode	
Sample	Used powder material	A/B ratio	Thickness (μm)	Used powder material	Porosity (%)
Cell-97	Ba_0.97_Zr_0.8_Yb_0.2_O_3−δ_	0.97	∼5	NiO-Ba_0.97_Zr_0.8_Yb_0.2_O_3−δ_	∼37
Cell-99	Ba_0.99_Zr_0.8_Yb_0.2_O_3−δ_	0.99	∼5	NiO-Ba_0.99_Zr_0.8_Yb_0.2_O_3−δ_	∼42

Figure 1 shows field emission scanning electron microscopy (FE-SEM) images of Cell-97 and Cell-99. In the FE-SEM images of the top surfaces of the BZYb20 electrolytes before cathode preparation (Figure 1a,b,d,e), although the surface morphologies of the BZYb20 electrolytes of Cell-97 and Cell-99 differed slightly, both BZYb20 electrolytes were well densified with similar grain sizes of approximately 1–2 μm. Cross-sectional FE-SEM images show the microstructure of the component layers of Cell-97 and Cell-99 (Figure 1c,f). Note that the FE-SEM observations of the cross-sectional microstructure of the PCFCs were carried out after the electrochemical measurements of the PCFCs using H_2_ fuel, and thus, the NiO in the anodes was reduced to Ni. In both the PCFCs, a dense BZYb20 electrolyte approximately 5 μm thick was formed on a porous Ni-BZYb20 anode. The anode microstructures of the two PCFCs were similar as far as observed by FE-SEM (Figure S2). An LBC-BZYb10 cathode was also deposited on the BZYb20 electrolyte without delamination. Because the LBC-BZYb10 cathode was prepared by using nanocomposite particles synthesized via spray pyrolysis, the cathode microstructure was very fine (Figure S3). In summary, the FE-SEM observations suggest that both PCFCs have nearly similar microstructures. Furthermore, to investigate the anode microstructure in more detail, we measured the porosity and pore size of the Ni-BZYb20 anodes of the PCFCs by mercury porosimetry. As a result, the open porosities of Cell-97 and Cell-99 were approximately 37% and 42%, respectively. In our previous work, we had evaluated the dependence of cell performance on its open porosity in SOFCs. In the case of SOFCs with anode open porosities of approximately 34% and 44%, the difference in anode concentration overpotential was less than 30 mV at a current density of 2 A cm^–2^, suggesting that the effect on cell performance is limited.^35^ Moreover, the anode concentration overpotential in PCFCs should be further reduced because no steam production occurs at the anode side. The pore size measurements revealed that the Ni-BZYb20 anodes of both PCFCs had two sizes of pores, namely, small pores of 0.21 μm and large pores of 0.55 μm for Cell-97 and small pores of 0.15 μm and large pores of 0.59 μm for Cell-99 (Figure S4). Although the porosity of Cell-99 was slightly higher than that of Cell-97, these characterizations confirm that both PCFCs have similar microstructures.

**Figure 1:**
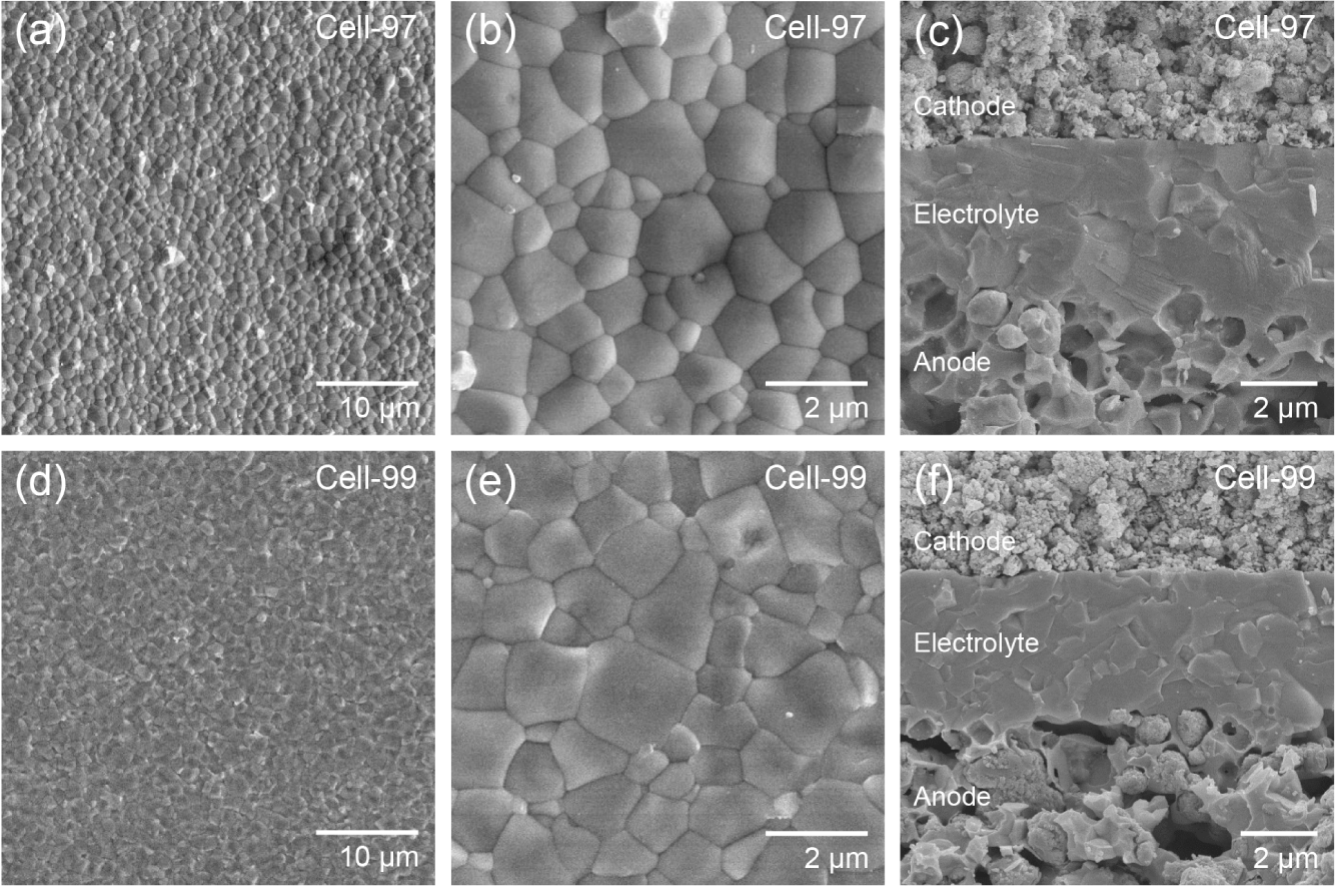
FE-SEM images of anode-supported PCFCs: (a, b) top surface of the BZYb20 electrolyte of Cell-97, (c) cross-section of Cell-97, (d, e) top surface of the BZYb20 electrolyte of Cell-99, and (f) cross-section of Cell-99.

To determine the element distribution on the BZYb20 electrolyte surfaces of Cell-97 and Cell-99, we used scanning electron microscopy (SEM) and energy-dispersive X-ray spectroscopy (EDX). In addition, the cation concentration over the entire surface of the BZYb20 electrolytes was measured by X-ray fluorescence (XRF). The A/B ratio of the BZYb20 electrolytes of PCFCs was slightly decreased compared with that of the BZYb20 raw powder materials, i.e., from 0.97 to 0.95 for Cell-97 and from 0.99 to 0.96 for Cell-99 (Table S1). Although perfect quantitative analysis might be difficult in our XRF analysis, we could reveal that the A/B ratio of the BZYb20 electrolyte of Cell-99 was higher than that of Cell-97 even with the PCFC electrolyte state via high-temperature cosintering. A small amount of Ni was also detected in both PCFCs because Ni diffused from the NiO-BZYb20 anodes to the BZYb20 electrolytes during the cosintering process. Figure 2 shows SEM images of the top surface view of the BZYb20 electrolytes and EDX mappings of the elements constituting the BZYb20 electrolytes, namely, Zr, Ba, and Yb. In the EDX mapping, the BZYb20 electrolytes of Cell-97 and Cell-99 exhibited different element distributions, i.e., a heterogeneous element distribution for Cell-97 and a homogeneous element distribution for Cell-99. On the BZYb20 electrolyte surface of Cell-97, although Zr and Ba were overlapped, Yb was separated from Ba and Zr, suggesting that the segregation of Yb_2_O_3_ occurred (Figure 2b–d). In contrast, on the BZYb20 electrolyte surface of Cell-99, all of the constituent elements of Zr, Ba, and Yb were uniformly distributed (Figure 2f–h). The different electrolyte surface conditions of Cell-97 and Cell-99 were mainly due to the difference in the A/B ratio of the BZYb20 raw powder materials used for these PCFCs. In addition, a relatively high-temperature sintering of Cell-97 (1475 °C) compared with that of Cell-99 (1430 °C) might promote Ba evaporation from the BZYb20 electrolyte. Note that Zr was detected in the same parts as Ba in the EDX observation for Cell-97 (Figure 2b,c), suggesting that the dopant cation of Yb^3+^ is preferable to phase-separate from the BZYb20 lattice. The segregation of Yb_2_O_3_ in Cell-97 leads to deterioration of the proton conductivity of the BZYb20 electrolyte due to a decrease in the amount of dopant cation Yb^3+^ and also leads to creation of inactive Yb_2_O_3_ areas on the BZYb20 electrolyte surface.^29–32^ As a result, not only the electrolyte ohmic resistance but also the cathode polarization resistance might increase in Cell-97.^33,36^ In the present work, although slightly Ba-deficient BZYb20 powders were used for the fabrication of Cell-99 as well as Cell-97 to avoid the formation of BaCO_3_, the BZYb20 electrolyte of Cell-99 exhibited a homogeneous element distribution without Yb_2_O_3_ segregation. This result indicates that an electrolyte surface capable of forming a good interface with a cathode was obtained in Cell-99.

**Figure 2:**
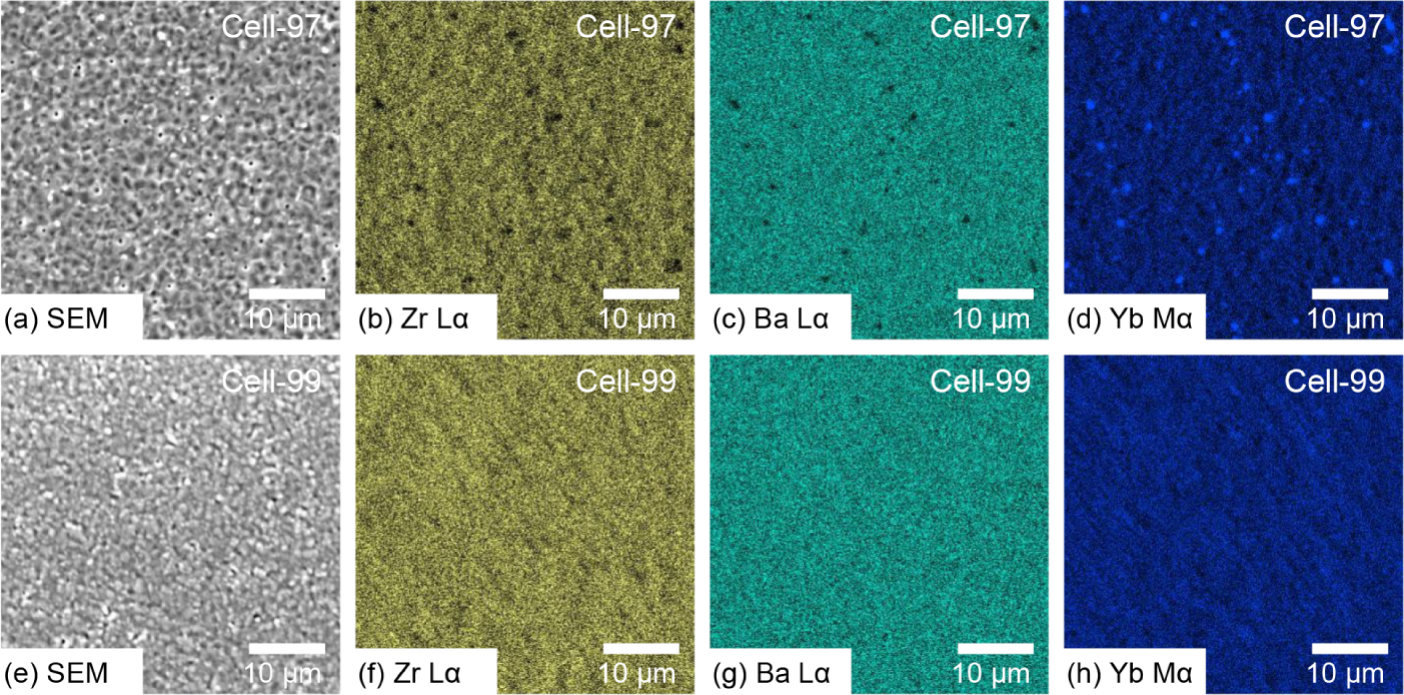
SEM-EDX images of BZYb20 electrolyte surfaces of anode-supported PCFCs: (a) SEM image of Cell-97, (b) Zr Lα mapping of Cell-97, (c) Ba Lα mapping of Cell-97, (d) Yb Mα mapping of Cell-97, (e) SEM image of Cell-99, (f) Zr Lα mapping of Cell-99, (g) Ba Lα mapping of Cell-99, and (h) Yb Mα mapping of Cell-99.

Figure 3a shows the X-ray diffraction (XRD) patterns for the top surfaces of the BZYb20 electrolytes before cathode preparation for Cell-97 and Cell-99. As a result, Yb_2_O_3_ peaks were observed in Cell-97 but not in Cell-99. This result corresponds to the SEM-EDX results (Figure 2). No peaks corresponding to BaCO_3_ were observed in any of these PCFCs. In addition, all the peaks in Cell-97 were slightly on the higher angle side than those in Cell-99, indicating a smaller lattice constant of the BZYb20 electrolyte of Cell-97 than that of Cell-99. We confirmed the formation of Yb_2_O_3_ in Cell-97 and different lattice constants 4.22178(3) Å of Cell-97 and 4.22445(4) Å of Cell-99 by Rietveld refinement (Figure S5). Figure 3b shows the BZYb20 raw powder materials used for fabrication of Cell-97 and Cell-99, namely, Ba_0.97_Zr_0.8_Yb_0.2_O_3−δ_ and Ba_0.99_Zr_0.8_Yb_0.2_O_3−δ_, respectively, for comparison with the XRD patterns for the BZYb20 electrolytes of PCFCs. Both XRD patterns for the BZYb20 raw powder materials had small peaks for BaCO_3_ along with the peaks for BZYb20, indicating that a small amount of BaCO_3_ was present in the raw powder state, even with the Ba-deficient chemical composition of BZYb20. In addition, although small peaks for Yb_2_O_3_ were observed in both powder materials, they were not as clear as those of Cell-97. From these results, the formation and segregation of Yb_2_O_3_ in Cell-97 were considered to occur during the high-temperature cosintering process (1475 °C). A low Ba-deficient BZYb20 and a low cosintering temperature as in the case of Cell-99 are key factors in suppressing the formation and segregation of Yb_2_O_3_.

**Figure 3:**
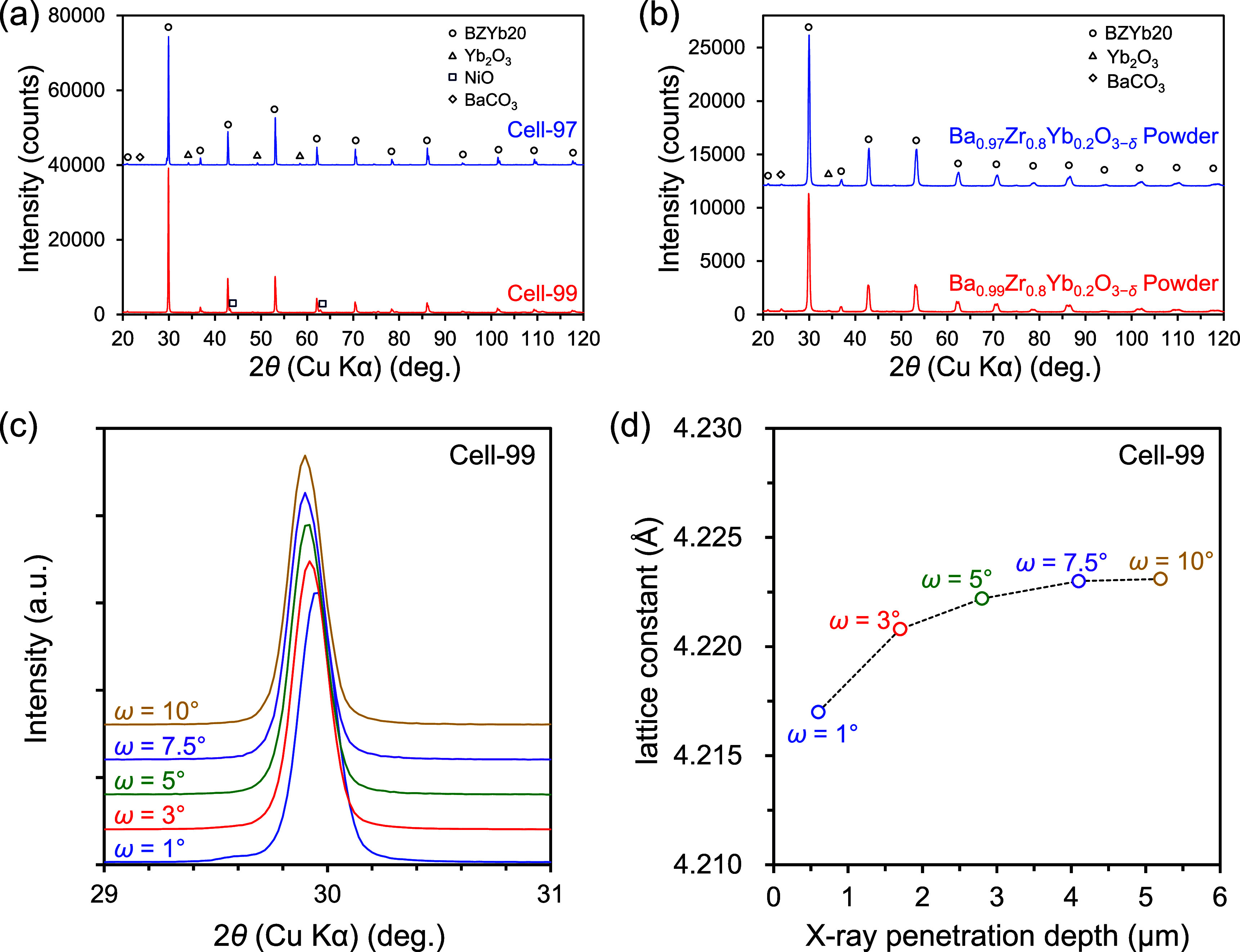
XRD patterns for BZYb20 electrolytes and powders measured with Cu Kα radiation at room temperature in ambient air: (a) BZYb20 electrolyte of Cell-97 and Cell-99 and (b) raw powder materials of Ba_0.97_Zr_0.8_Yb_0.2_O_3−δ_ used for Cell-97 and Ba_0.99_Zr_0.8_Yb_0.2_O_3−δ_ used for Cell-99. (c) BZYb20 electrolyte of Cell-99 by thin-film XRD analysis with incident angle ω of the X-ray fixed between 1° and 10°, and (d) lattice constants of the BZYb20 electrolyte of Cell-99 as a function of X-ray penetration depth.

To characterize in more detail the thin-film BZYb20 electrolytes on the PCFCs, we conducted thin-film XRD analysis with the incidence angle ω of the X-rays fixed between 1° and 10° (Figure S6). Figure 3c shows the XRD patterns for the top surfaces of the BZYb20 electrolytes of Cell-99 measured with the thin-film XRD analysis, and Figure 3d shows the depth dependence of the lattice constant calculated from the XRD patterns at each ω for the BZYb20 electrolyte of Cell-99. The lattice constant decreased with decreasing X-ray penetration depth, indicating that the lattice constant near the electrolyte surface became smaller. Factors determining the lattice constant of the BZYb20 electrolyte include the amount of hydrated proton, the amount of diffused Ni, and the amount of Ba on the A-site. Because the lattice constant decreases with a decreasing amount of Ba,^29^ the Ba evaporation from the BZYb20 electrolyte surface is the main reason for the decrease in the lattice constant.

### 2.2 Performance of PCFCs with Different BZYb20 Electrolytes

Electrochemical performance of Cell-97 and Cell-99 was evaluated in a temperature range of 500–700 °C using 3% humidified H_2_ as fuel and 3% humidified O_2_–N_2_ (21:79 vol %) as oxidant. Figure 4 shows the current–voltage (*I*–*V*) characteristics and the corresponding power densities for Cell-97 and Cell-99, and Table S2 lists the detailed values of the results. The power densities of Cell-99 were higher than those of Cell-97; for example, the maximum power density (MPD) at 600 °C was 1.306 W cm^–2^ for Cell-99, which was approximately 1.7 times higher than 0.787 W cm^–2^ for Cell-97. In terms of the open-circuit voltage (OCV), both PCFCs exhibited almost the same values. The measured OCVs were lower than the theoretical OCVs (Table S2) because BZYb20 intrinsically has a slight hole conductivity.^37–43^ These OCVs were very consistent with the calculated results reported in our previous work, where the OCVs of PCFCs with BZYb20 electrolyte were estimated by a numerical simulation method considering hole conduction.^16^ The hole conductivity of an electrolyte leads to current leakage in PCFCs, which affects the energy-conversion efficiency. The OCV is therefore an important indicator for evaluating the cell performance of PCFCs. Although for both PCFCs the measured OCVs improved with decreasing operating temperature, these OCVs were still lower than the theoretical OCVs, indicating that a slight current leakage occurred even at low temperatures around 500 °C. The OCV can be improved by increasing the electrolyte thickness and/or by using an additional functional layer with the electrolyte.^44,45^ However, these approaches might lower the power density because they affect the output external current by the proton conductivity and the leakage current by the hole conductivity in PCFCs. In essence, reducing the hole conductivity in proton-conducting electrolytes and suppressing current leakage in PCFCs are worldwide challenges in current PCFC development. We too will continue to investigate this important issue. In terms of durability, Cell-99 was also superior to Cell-97 (Figure S7). Although the durability tests were conducted for a short-term (approximately 50 h), Cell-99 showed stable operation with almost no degradation. These results indicate that the surface condition of the electrolyte affects not only power density but also durability of PCFCs.

**Figure 4:**
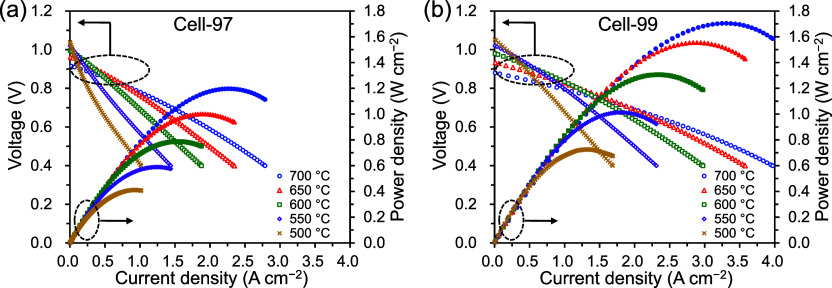
*I*–*V* characteristics and corresponding power densities at 500–700 °C using 3% humidified H_2_ as fuel and 3% humidified O_2_–N_2_ (21:79 vol %) as oxidant for (a) Cell-97 and (b) Cell-99.

We compared the MPDs for Cell-99 in the present work and for state-of-the-art anode-supported cells, including both SOFCs and PCFCs, as a benchmark (Table S3).^46–56^ Figure 5 clearly indicates the excellent performance of Cell-99, which is as high as that of state-of-the-art cells. In the temperature range below 600 °C, the MPDs of Cell-99 were higher than those of any SOFCs. Moreover, the MPDs of Cell-99 were comparable to those of PCFCs with Ce-containing Ba­(Zr,Ce)­O_3_-based electrolytes. The MPD of Cell-99 at 650 °C was slightly inferior to that of other top-level PCFCs because the hole conductivity of BZYb20 at 650 °C was relatively higher than that of Ba­(Zr,Ce)­O_3_-based electrolytes; below 600 °C, the hole conductivity of BZYb20 decreased, resulting in higher MPDs of Cell-99 compared with those of other PCFCs.^25^ It is noteworthy that Cell-99 achieves MPDs much higher than those of previously reported PCFCs with Ce-free BaZrO_3_-based electrolytes. The MPDs of Cell-99 were the highest values in PCFCs with BaZrO_3_-based electrolytes, to the best of our knowledge. The results of this comparison here indicate that with an adequate chemical composition of BZYb20 and an optimal fabrication process, a high-performance PCFC can be realized even without the use of costly materials and/or special processes.

**Figure 5:**
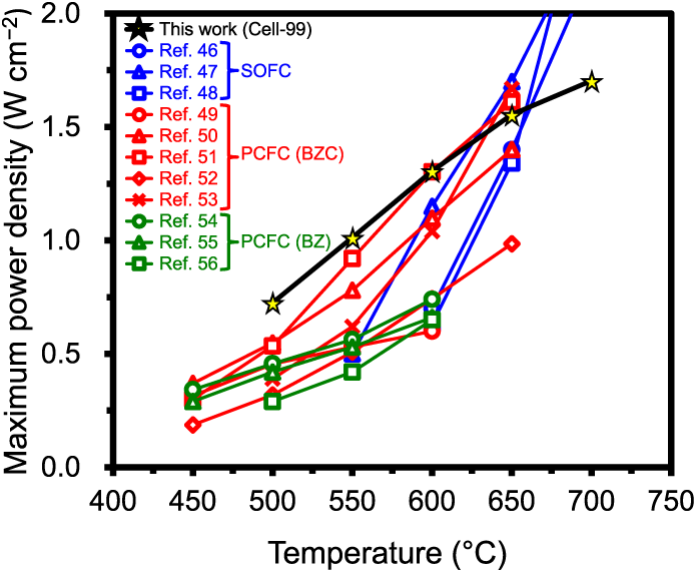
Comparison of maximum power densities among Cell-99 in this work and previously reported anode-supported SOFCs and PCFCs. Ba­(Zr,Ce)­O_3_-based and BaZrO_3_-based electrolytes denoted as BZC and BZ, respectively.^46–56^ .

Figure 6a,b shows the Nyquist plots of the impedance spectra at 0.85 V for Cell-97 and Cell-99, respectively. Note that the electrochemical impedance spectra (EIS) measurements were carried out under an operating condition of 0.85 V here because EIS measurements under the OCV condition might evaluate the cell resistance of a PCFC to a lower value than the true value (Figure S8). The hole conductivity of proton-conducting electrolytes including BZYb20 generally increases with increasing oxygen partial pressure.^14,26,38^ Under operating conditions in PCFCs, the oxygen partial pressure at the cathode side decreases by electrochemically produced H_2_O, thus reducing the effect of the hole conductivity of a proton-conducting electrolyte on EIS results.^27^

**Figure 6:**
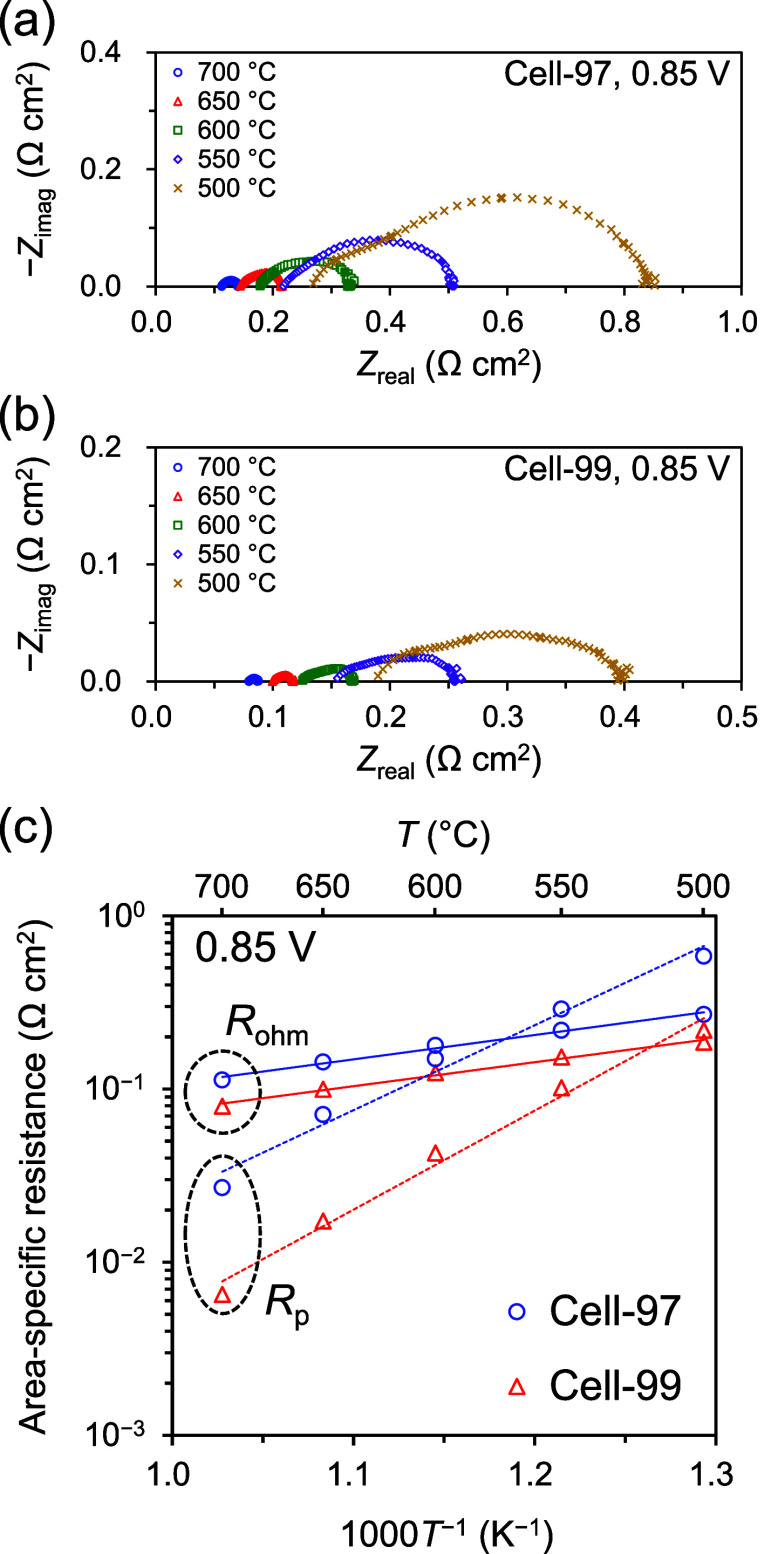
EIS results of PCFCs at 500–700 °C using 3% humidified H_2_ as fuel and 3% humidified O_2_–N_2_ (21:79 vol %) as oxidant under operating condition of 0.85 V: (a) Nyquist plots for Cell-97, (b) Nyquist plots for Cell-99, and (c) ASRs of *R*_ohm_ and *R*_p_ estimated by simply reading Nyquist plots as a function of operating temperature.

Figure 6c shows the area-specific resistances (ASRs) of the ohmic resistance, *R*_ohm_, and the electrode polarization resistance, *R*_p_, as a function of the operating temperature for Cell-97 and Cell-99. The ASRs were estimated here by simply reading the Nyquist plots (Figure 6a,b); *R*_ohm_ was determined from the intercept on the real axis at the higher frequency side of the Nyquist plots, and *R*_p_ was determined from the difference between the two intercepts on the real axis at the lower and higher frequency sides. In general, ASR estimation by simply reading the Nyquist plots might result in slight errors in some cases. In Figure 6c, both *R*_ohm_ and *R*_p_ showed an Arrhenius-type temperature dependence. We therefore consider that the separation of *R*_ohm_ and *R*_p_ is basically appropriate. The activation energy for *R*_ohm_ was the same value (27 kJ mol^–1^) for both the PCFCs, both of which correspond to that for typical proton conduction in perovskite-type proton-conducting electrolytes,^22^ indicating that *R*_ohm_ was mainly due to the resistance of the BZYb20 electrolytes in both PCFCs. Over the entire temperature range (500–700 °C), Cell-99 exhibited lower resistances than Cell-97 for both *R*_ohm_ and *R*_p_. The difference in *R*_ohm_ was considered to be due to the difference in the chemical composition of the BZYb20 electrolytes of Cell-97 and Cell-99.^29^ Notably, *R*_p_ of Cell-99 was significantly reduced compared to that of Cell-97. Although *R*_p_ here includes the polarization resistances of both the cathode and anode, the observed difference in *R*_p_ can be mainly attributed to the cathode polarization resistance because the cathode polarization resistance is generally much higher than the anode polarization resistances in PCFCs.^57–59^ These results suggest that optimal electrolyte surface conditions, such as in Cell-99, can improve the cathode performance in PCFCs even with the same cathode material.

### 2.3 Effect of BZYb20 Electrolyte Surface Condition on Cathode Performance

To investigate in more detail the effect of the surface condition of the BZYb20 electrolyte on cell performance, we compared the impedance spectra measured at a representative temperature of 600 °C for Cell-97 and Cell-99. Figure 7a,b shows the Nyquist plots and the Bode plots of the impedance spectra, respectively. The Nyquist plots clearly show that the apparent arc size of Cell-99 was much smaller than that of Cell-97, suggesting a higher electrode performance of Cell-99 (Figure 7a). In the Bode plots, the highest peaks occurred in the same frequency range of 10–100 Hz for both of these PCFCs, suggesting that the rate-determining step for the electrode electrochemical reaction did not change in either of these PCFCs (Figure 7b).

**Figure 7:**
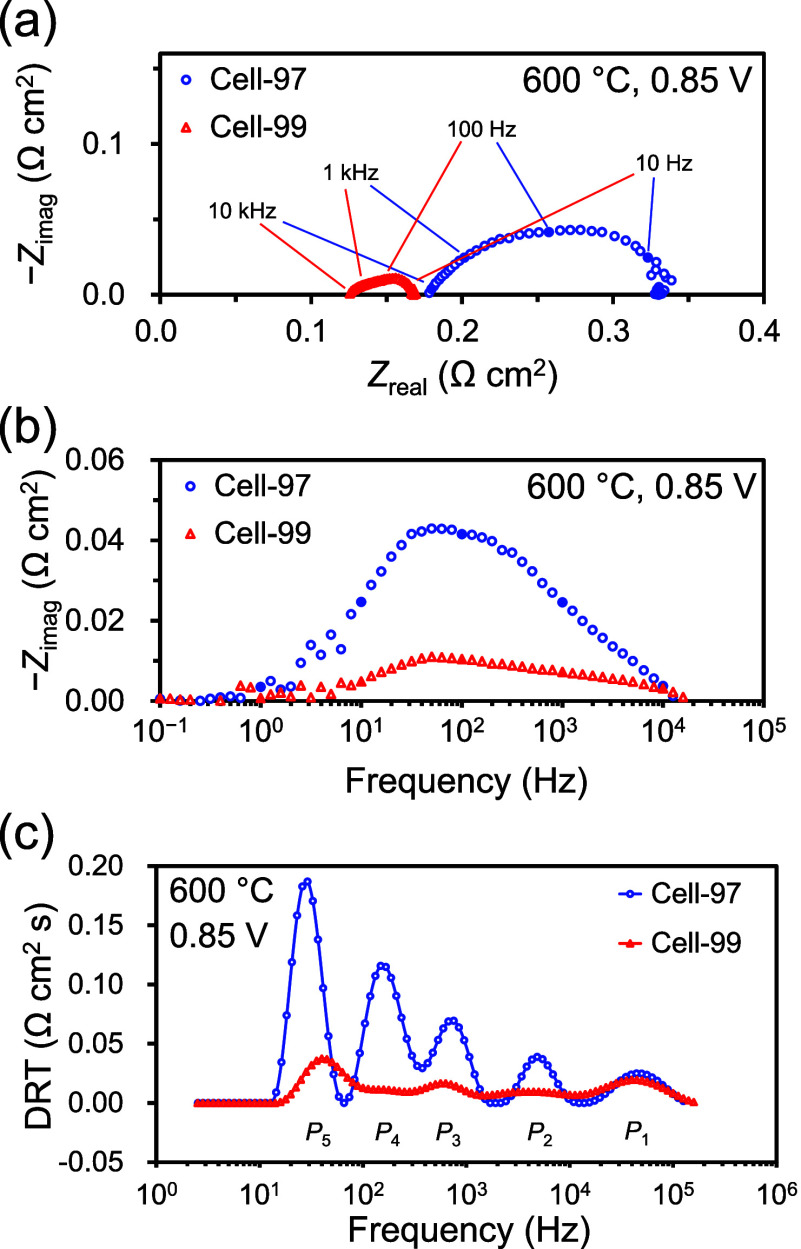
Comparison of EIS results between Cell-97 and Cell-99 at 600 °C using 3% humidified H_2_ as fuel and 3% humidified O_2_–N_2_ (21:79 vol %) as oxidant under operating condition of 0.85 V: (a) Nyquist plots, (b) Bode plots, and (c) DRT spectra.

*R*_p_ estimated from the Nyquist plots includes the polarization resistances of both the cathode and anode. To extract the cathode polarization from *R*_p_, we analyzed the impedance spectra at 600 °C by deconvoluting the impedance spectra using the distribution of relaxation times (DRT) analysis.^60,61^ As shown in Figure 7c, five DRT peaks were detected from the impedance spectra for Cell-97 and Cell-99. These DRT peaks are denoted as *P*_1_, *P*_2_, *P*_3_, *P*_4_, and *P*_5_ in order of high to low frequency ranges. The DRT peaks for Cell-99 tended to be lower in height than those for Cell-97, and this tendency was especially noticeable for the DRT peaks in low-frequency ranges, namely, *P*_3_, *P*_4_, and *P*_5_.

By employing complex nonlinear least-squares (CNLS) fitting using the DRT analysis results and the equivalent circuit model shown in Figure 8a, we estimated the detailed ASRs attributed to electrochemical reaction processes in Cell-97 and Cell-99. In the equivalent circuit model, *L*, *R*, and *C* represent the inductance, resistance, and capacitance, respectively. The fitting curves and detailed equivalent circuit model parameters are also shown in Figure S9 and Table S4, respectively. We were able to fit the experimental plots with reasonable parameters. Figure 8b shows the estimated ASRs of each PCFC. The ASRs of *R*_1_–*R*_5_ were the resistances corresponding to the DRT peaks of *P*_1_–*P*_5_, attributed to the various electrode polarization processes in the PCFCs. In general, it is difficult to know the physicochemical origins to which each DRT peak corresponds in a PCFC because current leakage complicates the EIS analysis. We have, however, determined the physicochemical origins for each DRT peak observed in PCFCs in previous works.^62,63^ The main results are as follows: *P*_1_ (10–100 kHz): physical impedance originating from grain boundaries, pores, and reaction products between the electrolyte and electrodes; *P*_2_ (0.1–10 kHz): hydrogen oxidation process at the anode; *P*_3_ (20–1000 Hz): steam production process at the cathode,; *P*_4_ (2–200 Hz): oxygen reduction process at the cathode; *P*_5–1_ (0.5–20 Hz): gas diffusion process in the electrodes; and *P*_5–2_ (0.02–10 Hz): nonstoichiometric oxygen variation process at the cathode/electrolyte interface. To simplify, *P*_1_, *P*_3_, *P*_4_, and *P*_5_ are related to the cathode polarization processes, while *P*_1_, *P*_2_, and *P*_5_ are related to anode polarization processes. Based on the above knowledge, we discuss the ASRs between Cell-97 and Cell-99 here.

**Figure 8:**
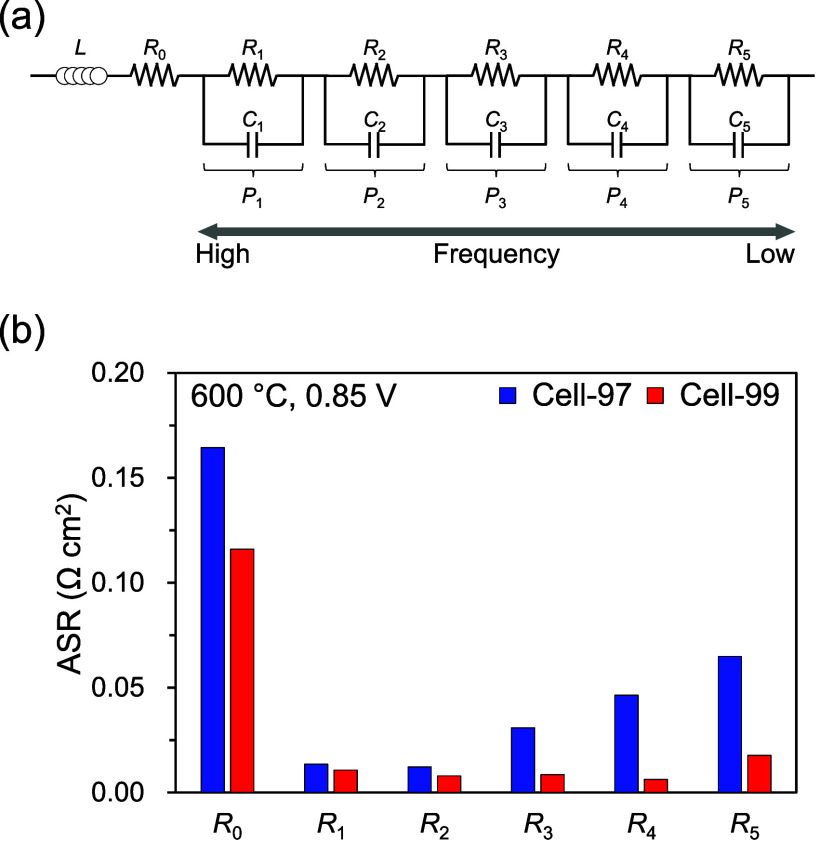
Resistance separation results of Cell-97 and Cell-99 at 600 °C using 3% humidified H_2_ as fuel and 3% humidified O_2_–N_2_ (21:79 vol %) as oxidant under operating condition of 0.85 V: (a) equivalent circuit model used for CNLS fitting and (b) ASRs separated by CNLS fitting with DRT analysis.

In comparison between Cell-97 and Cell-99, all of the ASRs (*R*_0_–*R*_5_) of Cell-99 were lower than those of Cell-97. Although *R*_0_ was refined by CNLS fitting, the resistance is attributed to basically the same process as *R*_ohm_, namely, the ohmic conduction process in the BZYb20 electrolytes. Thus, *R*_0_ had almost the same values as *R*_ohm_ in each PCFC. In Cell-97, *R*_3_, *R*_4_, and *R*_5_ due to cathode polarization were apparently high, whereas *R*_2_ due to anode polarization was the lowest in the ASRs of Cell-97, confirming that the cell performance of Cell-97 was dominated by the cathode polarization resistance similar to general PCFCs. Comparing each ASR between the two PCFCs, *R*_1_ of Cell-99 was slightly lower than that of Cell-97. We interpret that no clear difference was expected in *R*_1_ between the two PCFCs because *R*_1_ was relatively low compared with the other ASRs and might be affected by *R*_0_, which was a high ASR in a close frequency range. Then, *R*_2_ was also not significantly different between the two PCFCs, suggesting that almost equivalent anode performance could be achieved despite using the different BZYb20 raw powder materials and sintering at different temperatures. In Cell-99, which has the improved BZYb20 electrolyte with a relatively high A/B ratio, a large lattice constant, and no Yb_2_O_3_ segregation, the cathode polarization resistances of *R*_3_, *R*_4_, and *R*_5_ were significantly reduced compared with those of Cell-97. The cathode reaction processes related to *R*_3_, *R*_4_, and *R*_5_ occur on the double-phase boundary (DPB, cathode/gas) and/or triple-phase boundary (TPB, cathode/electrolyte/gas). As observed here, all the cathode polarization resistances depended on the electrolyte surface condition, suggesting the importance of the role of the TPB region on the cathode reactions in PCFCs. In addition, the electrolyte surface condition might have affected the cathode surface reactions at the DPB region, such as oxygen dissociation, adsorption, and surface diffusion.

The objective of the present work is to realize a high-performance PCFC and to reveal the effect of the electrolyte surface condition with respect to the chemical composition such as stoichiometry and element distribution on the cathode performance in PCFCs. The decreases in *R*_3_, *R*_4_, and *R*_5_ observed here evidently indicate that the cathode performance strongly depends on the electrolyte surface condition in PCFCs. In summary, we revealed that the electrolyte surface condition affects various cathode polarization processes, such as the cathode steam production process, cathode oxygen reduction process, and interfacial nonstoichiometric oxygen variation process, which are the physicochemical origins for *R*_3_, *R*_4_, and *R*_5_, respectively. An optimal electrolyte surface condition enhances cathode performance, resulting in realizing high-performance PCFCs.

## 3 Conclusions

To improve the power density in PCFCs with chemically stable Ce-free BaZrO_3_-based electrolytes, we investigated anode-supported PCFCs using BZYb20 electrolytes, focusing on the effect of the surface condition of the BZYb20 electrolytes on cathode performance. Two PCFCs, namely, Cell-97 and Cell-99, were fabricated using two BZYb20 powders with different A/B ratios, i.e., Ba_0.97_Zr_0.8_Yb_0.2_O_3−δ_ and Ba_0.99_Zr_0.8_Yb_0.2_O_3−δ_, respectively. Regardless of the BZYb20 raw powder, almost similar microstructures were achieved in both PCFCs, with a dense thin BZYb20 electrolyte (∼5 μm thick) on the porous Ni-BZYb20 anode (open porosity of ∼37% in Cell-97 and ∼42% in Cell-99), by optimizing the cosintering process, i.e., 1475 °C for Cell-97 and 1430 °C for Cell-99. The surfaces of the BZYb20 electrolytes of Cell-97 and Cell-99 had different element distributions; Cell-97, which was sintered at a relatively high temperature, exhibited a heterogeneous element distribution with Yb_2_O_3_ segregation, whereas Cell-99 exhibited a homogeneous element distribution. In addition, XRD analysis revealed the formation of Yb_2_O_3_ on the BZYb20 electrolyte of Cell-97. These characterization results indicate that Cell-97 and Cell-99 had almost similar microstructures but different electrolyte surface conditions. Electrochemical performance of Cell-99 was exceptionally high and superior to that of Cell-97. The MPD of Cell-99 reached 1.306 W cm^–2^ at 600 °C, which was higher than the MPD (0.787 W cm^–2^) of Cell-97. Moreover, the MPDs of Cell-99 were as high as those of various state-of-the-art cells, including SOFCs and PCFCs with Ba­(Zr,Ce)­O_3_-based electrolytes, and were particularly the highest in PCFCs with Ce-free BaZrO_3_-based electrolytes. The DRT analysis and CNLS fitting revealed that the cathode polarization resistance of Cell-99 was much lower than that of Cell-97. These results indicate that a high-performance PCFC can be achieved with a cathode whose performance is enhanced by forming it on an electrolyte surface with an optimized chemical composition and fabrication process. As described above, the present work revealed the impact of the electrolyte surface condition on cathode polarization. Currently, we are planning next work focusing on interface engineering, including electrode materials. In future work, we would like to report on PCFCs that achieve even higher power density and durability, contributing to realize practical, high-efficiency applications.

## 4 Experimental Section

### 4.1 Fabrication of Anode-Supported PCFCs

Anode-supported PCFCs were fabricated using two BZYb20 powders with different A/B ratios, i.e., Ba_0.97_Zr_0.8_Yb_0.2_O_3−δ_ (5.7 m^2^ g^–1^ specific surface area, Sakai Chemical Industry Co.) and Ba_0.99_Zr_0.8_Yb_0.2_O_3−δ_ (4.9 m^2^ g^–1^ specific surface area, Sakai Chemical Industry Co.). The PCFCs fabricated using Ba_0.97_Zr_0.8_Yb_0.2_O_3−δ_ and Ba_0.99_Zr_0.8_Yb_0.2_O_3−δ_ were denoted here as Cell-97 and Cell-99, respectively.

The anode material for Cell-97 and Cell-99 was a mixture of the respective BZYb20 powder, NiO powder (3.3 m^2^ g^–1^ specific surface area, Sumitomo Metal Mining Co.), and graphite carbon (UF-G10, Resonac Holdings) in a weight ratio of 60:40:10. Two NiO-BZYb20 slurries were prepared by ball-milling these three powders with polyvinyl butyral binder, amine-based dispersant, and adipic acid-based plasticizer in a solvent mixture of toluene and 1-butanol for 48 h. The NiO-BZYb20 slurries were then tape-casted and dried in air. The obtained NiO-BZYb20 green sheets were laminated and hot-pressed at 60 °C at 15 MPa and then cut into 30 mm diameter pieces, resulting in NiO-BZYb20 green pieces.

Two BZYb20 slurries for the electrolyte were prepared by ball-milling each of the two BZYb20 powders with polyvinyl butyral binder, amine-based dispersant, and adipic acid-based plasticizer in a solvent mixture of ethanol and toluene. The BZYb20 slurries were spin-coated at 3000 rpm onto the NiO-BZYb20 green pieces. The pieces for fabricating Cell-97 and Cell-99 were cosintered at 1475 °C for 2 h in air and at 1430 °C for 3 h in air, respectively. These sintered half-cells were approximately 22 mm in diameter. The BZYb20 electrolyte and NiO-BZYb20 anode of the half-cells were approximately 5 μm and 0.6 mm in thickness, respectively.

A cathode was prepared using nanocomposite particles consisting of LBC and BZYb10 composites at a weight ratio of 50:50 synthesized via spray pyrolysis. The apparatus and preparation procedure of the spray pyrolysis process have been described elsewhere.^64^ In our process, two materials, LBC and BZYb10, were synthesized simultaneously, and thus, the nominal and resulting chemical compositions might not exactly match. The synthesized material, however, is called here as LBC-BZYb10 because our previous work successfully synthesized a composite material based on LBC and BZYb10.^16^ The LBC-BZYb10 nanocomposite particles were mixed with ethyl cellulose, dispersant, and plasticizer in α-terpineol solvent. The LBC-BZYb10 paste was screen-printed at 6 mm diameter on the BZYb20 electrolytes of the half-cells. After the paste was dried on a hot plate at 150 °C for 30 min in air, the cathode-printed half-cells were pressed at 300 MPa by using a cold isostatic press. Then, the LBC-BZYb10 cathode was obtained by sintering at 900 °C for 1 h in air, and its resulting thickness was approximately 5 μm.

### 4.2 Characterization

The microstructure of the PCFCs was observed by using FE-SEM (JSM-6330F, JEOL). The atomic concentration of Zr, Ba, and Yb of the BZYb20 electrolytes was analyzed using EDX equipped with SEM (JCM-6000Plus NeoScope, JEOL) and using XRF (ZSX Primus II, Rigaku). The crystal structure of the BZYb20 powders and the BZYb20 electrolytes of the PCFCs was determined using XRD (X’Pert Pro MPD, PANalytical) equipped with parallel-beam optics (having an incident focusing mirror, a diffracted graphite monochromator, and a proportional counter) with Cu Kα radiation at room temperature. The lattice constants were estimated by Rietveld refinement (RIETAN-FP). The porosity and pore size of the anode were measured by using mercury porosimetry (AutoPore IV 9520, Micromeritics).

### 4.3 Electrochemical Measurements

Electrochemical performance, namely, *I*–*V* characteristics and EIS of the PCFCs, was measured using a potentiostat/galvanostat with a frequency response analyzer (VSP-300, Biologic). In the electrochemical measurements, 3% humidified H_2_ was supplied as fuel to the anode at a flow rate of 100 mL min^–1^ and 3% humidified O_2_–N_2_ gaseous mixture (O_2_:N_2_ = 21:79 vol %) was supplied as oxidant to the cathode at a flow rate of 200 mL min^–1^. The *I*–*V* and EIS measurements were carried out at 500–700 °C. In EIS, the frequency range was 1 MHz to 0.1 Hz, and the applied voltage amplitude was 10 mV. The EIS results were deconvoluted by DRT analysis using Z-Assist software (Toyo Corporation) and by CNLS fitting with an equivalent circuit model using ZView software (Scribner Associates).

## Supplementary Material



## Notes

Cross-sectional FE-SEM image of overall Cell-99 as a representative anode-supported PCFC (Figure S1), cross-sectional FE-SEM images of Ni-BZYb20 anodes of PCFCs (Figure S2), cross-sectional FE-SEM image of LBC-BZYb10 cathode on Cell-99 (Figure S3), pore diameter distribution of Ni-BZYb20 anodes measured by mercury porosimetry (Figure S4), Rietveld refinement results of the XRD patterns measured with Cu Kα radiation at room temperature for BZYb20 electrolyte of PCFCs (Figure S5), schematic of thin-film XRD analysis with an incident angle ω of the X-ray fixed between 1° and 10° (Figure S6), short-term durability test results for PCFCs at 600 °C using 3% humidified H_2_ as fuel and 3% humidified O_2_–N_2_ (21:79 vol %) as oxidant under constant current density operation (Figure S7), Nyquist plots at 500–700 °C using 3% humidified H_2_ as fuel and 3% humidified O_2_–N_2_ (21:79 vol %) as oxidant under OCV condition for PCFCs (Figure S8), fitting curves at 600 °C using 3% humidified H_2_ as fuel and 3% humidified O_2_–N_2_ (21:79 vol %) as oxidant under operating condition of 0.85 V for PCFCs (Figure S9), cation concentrations of Ba, Zr, Yb, and Ni of BZYb20 electrolyte surface of PCFCs determined by XRF (Table S1), detailed MPDs and OCVs of PCFCs (Table S2), detailed components of previously reported SOFCs and PCFCs shown in Figure 5 (Table S3), and summary of CNLS fitting results with equivalent circuit model for PCFCs (Table S4) (PDF)
