# Supplementary material for: High-Performance Protonic Ceramic Fuel Cell with Ytterbium-Doped Barium Zirconate: Reducing Cathode Polarization by Improving Electrolyte Surface Condition
Source: ACS Appl Mater Interfaces. 2025 Jul 2;17(28):40639–49. doi: 10.1021/acsami.5c04806 (PMC12278254; doi:10.1021/acsami.5c04806)
Supplement: Supplementary file 1 [file am5c04806_si_001.pdf]

Supporting Information

High-Performance Protonic Ceramic Fuel Cell with Ytterbium-Doped Barium Zirconate: Reducing Cathode Polarization by Improving Electrolyte Surface Condition

Hiroyuki Shimada,* Konosuke Watanabe, Masaya Fujioka, Katsuhiro Nomura, Aman Sharma, Yuki Yamaguchi, Hirofumi Sumi, Yasunobu Mizutani

Innovative Functional Materials Research Institute, Department of Materials and Chemistry, National Institute of Advanced Industrial Science and Technology (AIST), 4-205 Sakurazaka, Moriyama-ku, Nagoya, Aichi 463-8560, Japan

* Corresponding author. E-mail: h.shimada@aist.go.jp

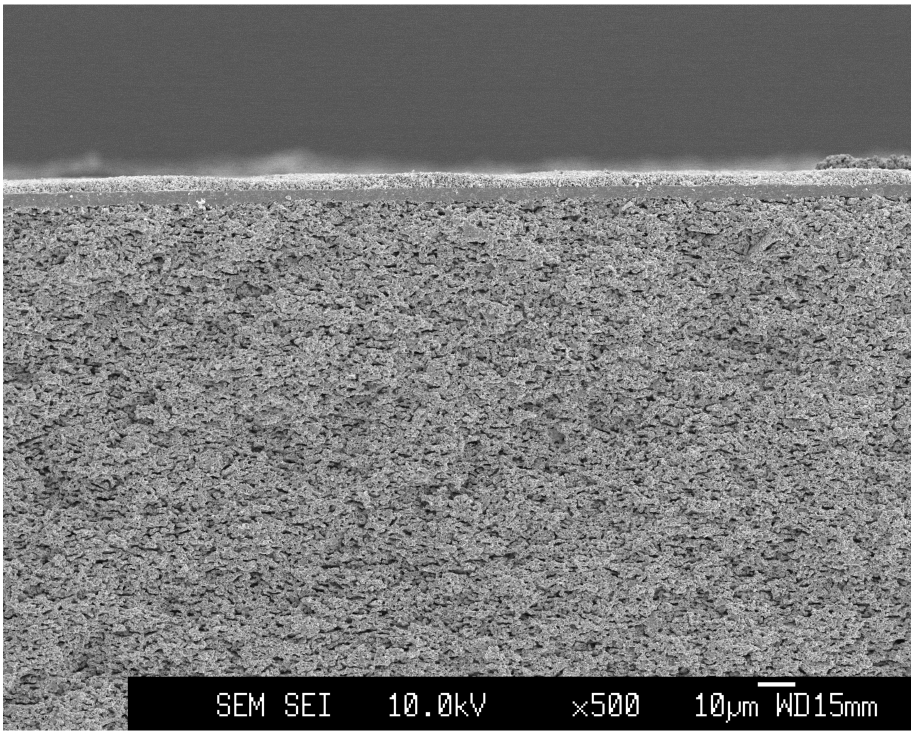

Figure S1

Cross-sectional FE-SEM image of overall Cell-99 as a representative anode-supported PCFC. Approximate thickness of each cell component was 5 μm for LBC-BZYb10 cathode, 5 μm for BZYb20 electrolyte, and 0.55 mm for Ni-BZYb20 anode.

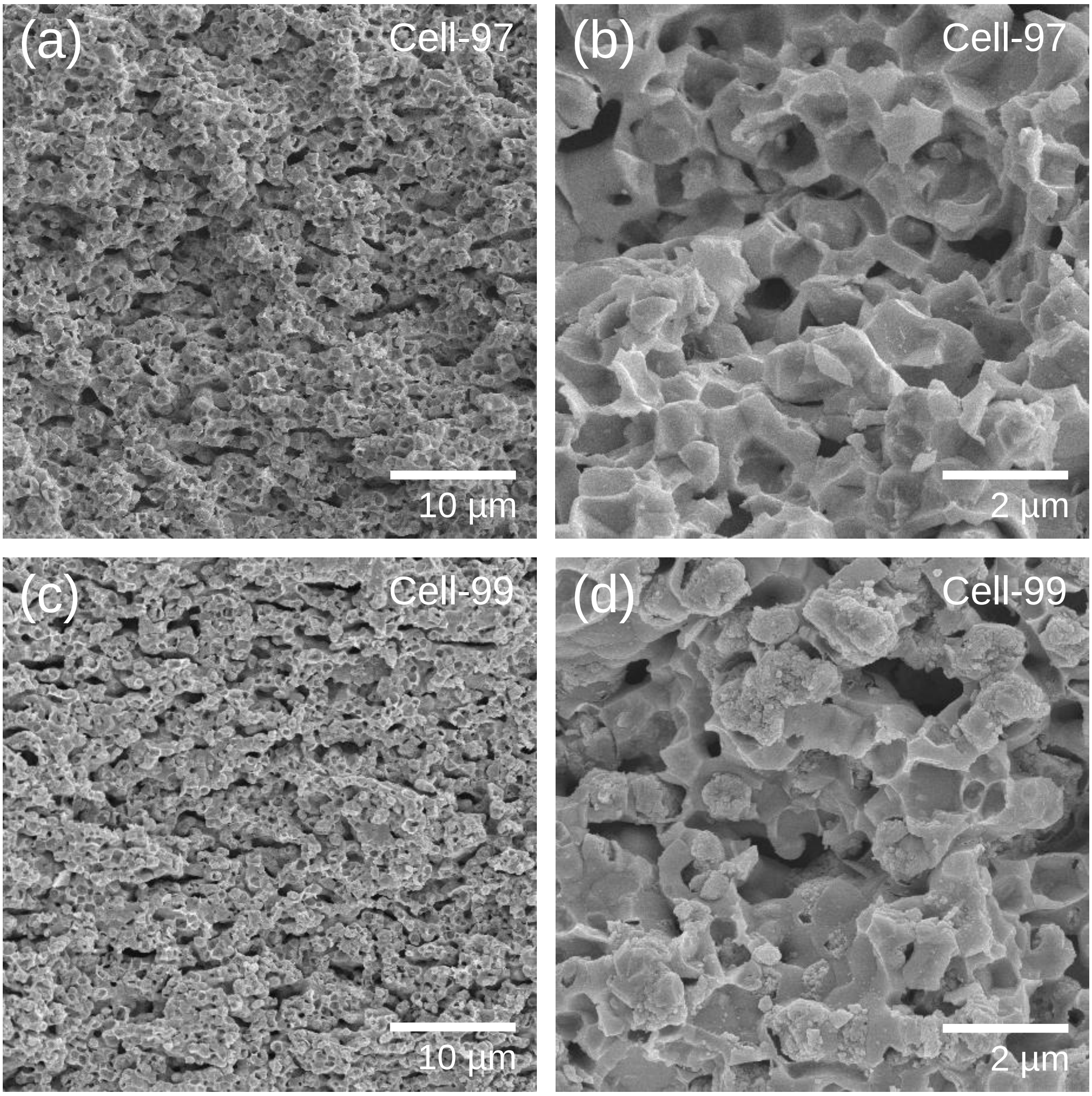

Figure S2

Cross-sectional FE-SEM images of Ni-BZYb20 anodes of (a, b) Cell-97 and (c, d) Cell-99.

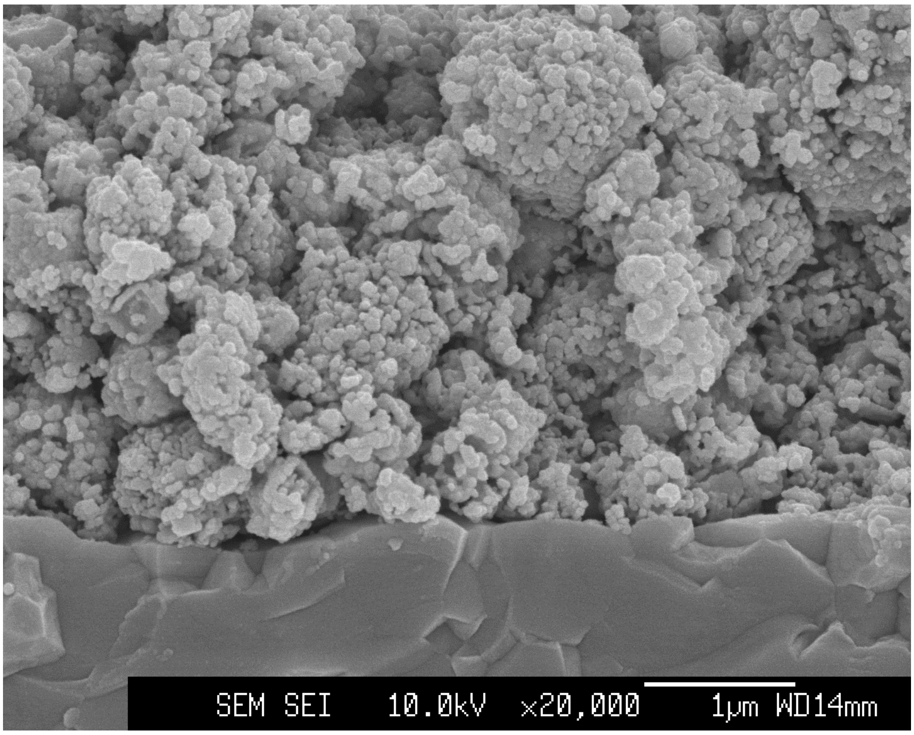

Figure S3.

Cross-sectional FE-SEM image of LBC-BZYb10 cathode on Cell-99. The cathode was prepared using LBC-BZYb10 nanocomposite particles synthesized via spray pyrolysis.

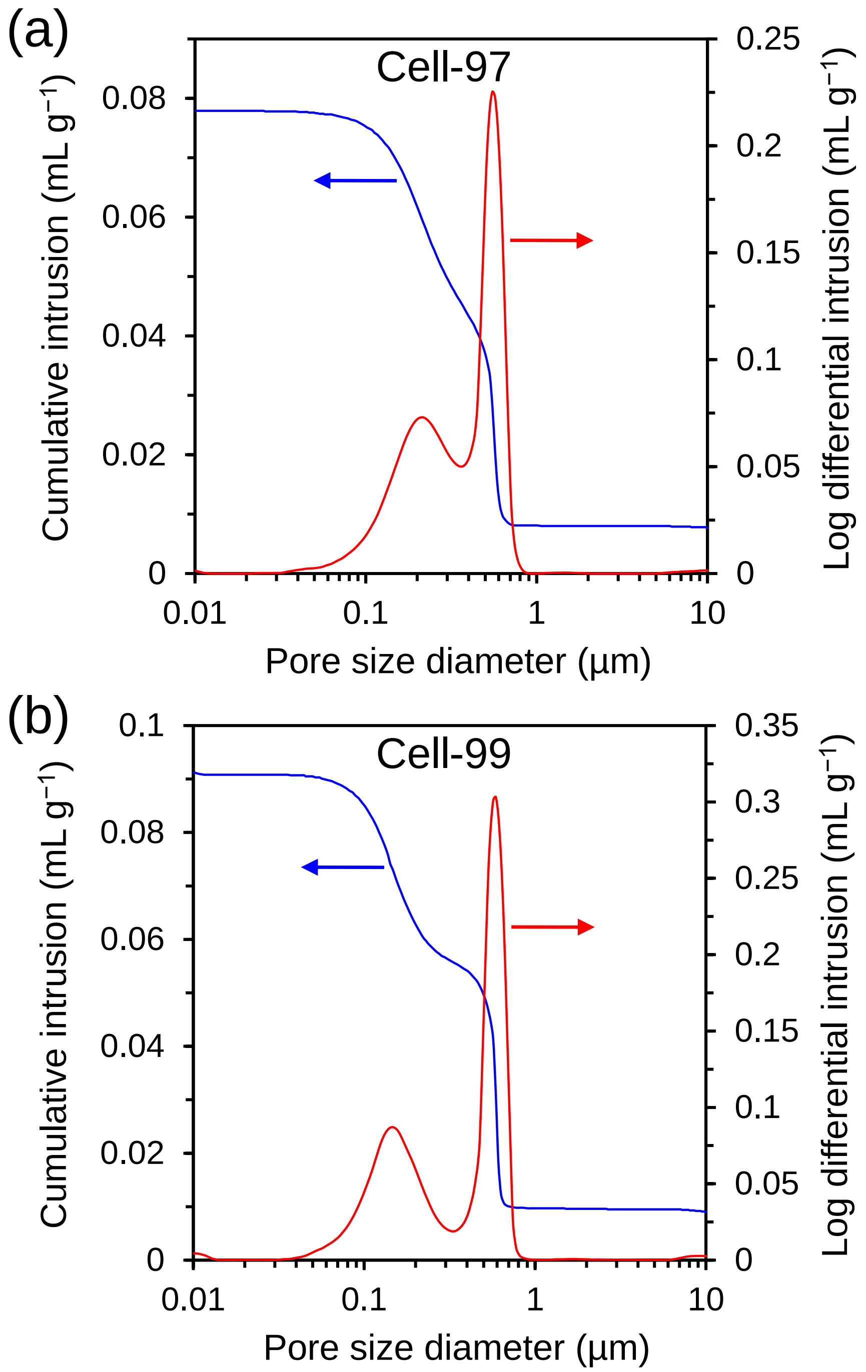

Figure S4

Pore diameter distribution of Ni-BZYb20 anodes measured by mercury porosimetry for (a) Cell-97 and (b) Cell-99. The pore size distribution had two peaks for each PCFC: 0.21 and 0.55 μm for Cell-97 and 0.15 and 0.59 μm for Cell-99.

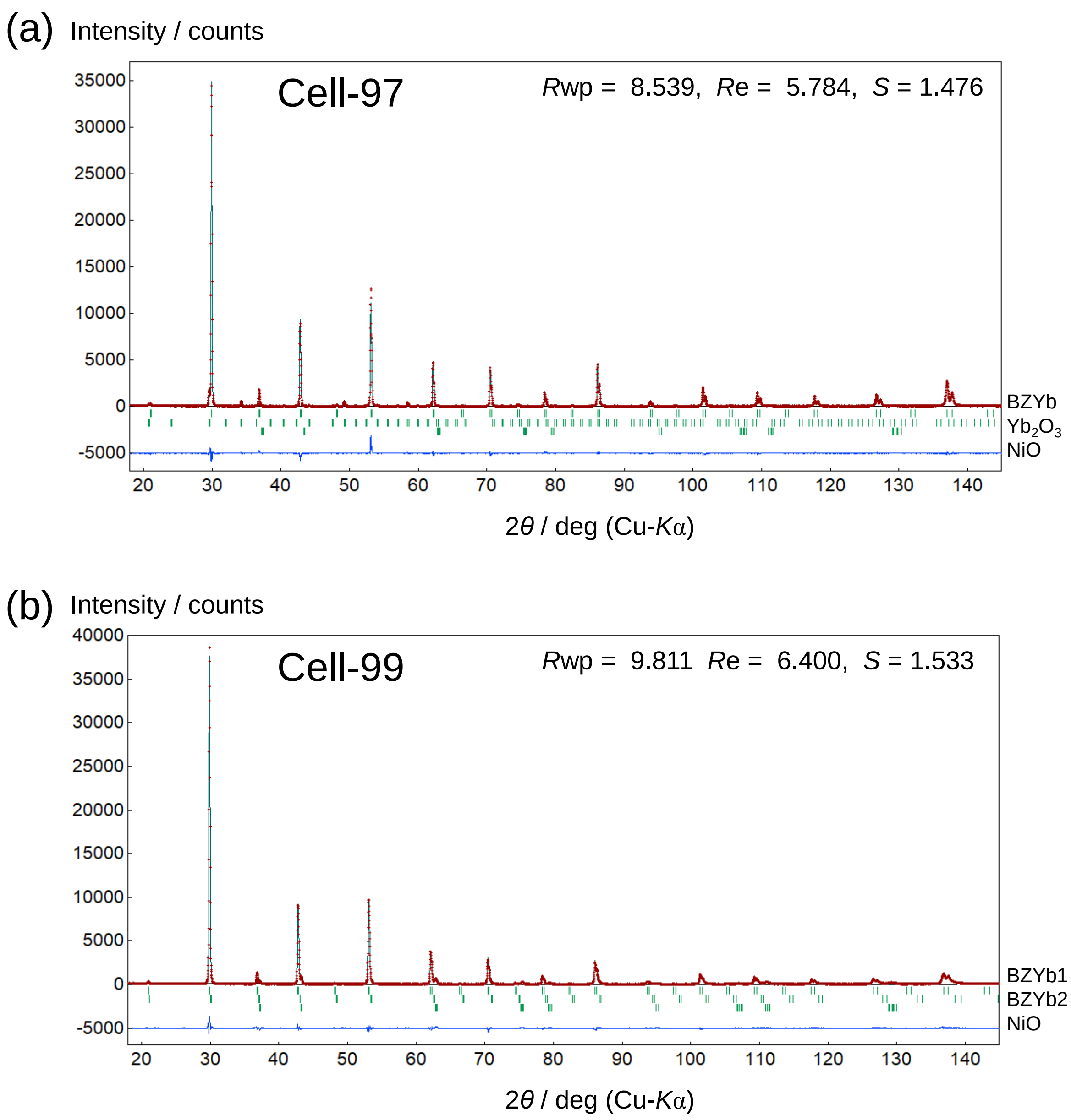

Figure S5

Rietveld refinement results of the XRD patterns measured with Cu Kα radiation at room temperature for BZYb20 electrolyte of (a) Cell-97 and (b) Cell-99. The goodness-of-fit indicator, *S*, of the Rietveld refinement was 1.476 for Cell-97 and 1.533 for Cell-99. The space group of the BZYb20 electrolyte was $Pm\bar{3}m$ for both Cell-97 and Cell-99. The lattice constant of the BZYb20 electrolyte was 4.22178(3) Å for Cell-97 and 4.22445(4) Å for Cell-99.

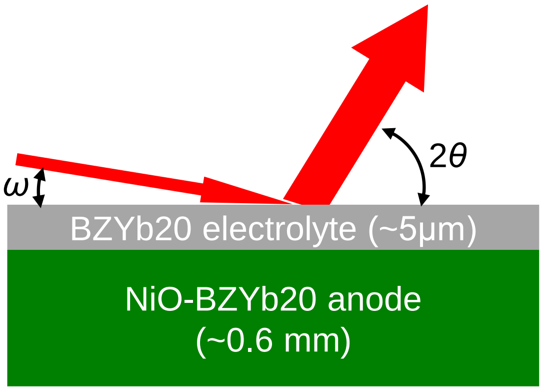

Figure S6

Schematic of thin-film XRD analysis with incident angle ω of the X-ray fixed between 1° and 10°. The X-ray penetration depth t was calculated using the following equation (S1):

$$t = \frac{4.61}{\mu} / \left(\frac{1}{\sin \omega} + \frac{1}{\sin (2\theta - \omega)} \right) \qquad \text{(S1)}$$

where t is the X-ray penetration depth (cm), μ is the linear absorption coefficient (cm^{-1}), ω is the incident angle (deg.), and θ is the diffraction angle (deg.).

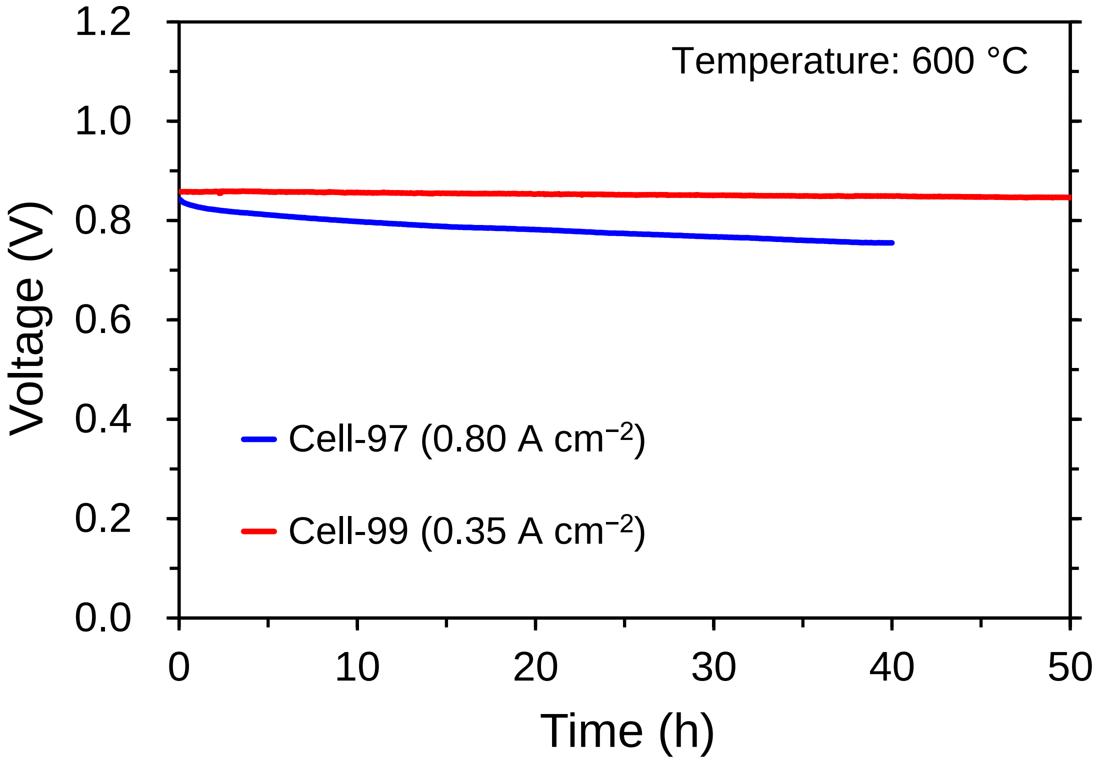

Figure S7

Short-term durability test results for PCFCs at 600 °C using 3% humidified H_2 as fuel and 3% humidified O_2–N_2 (21:79 vol%) as oxidant under constant current density operation.

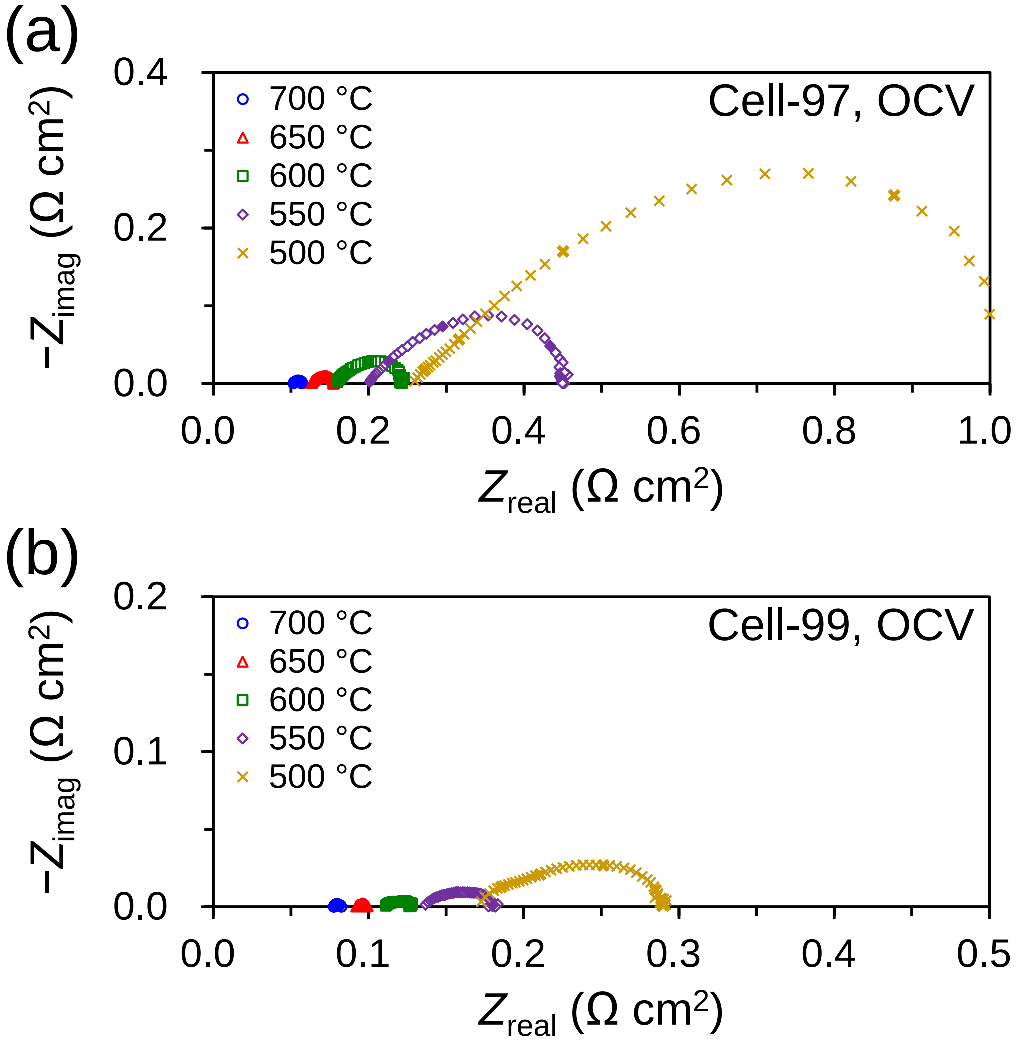

Figure S8

Nyquist plots at 500–700 °C using 3% humidified H_2 as fuel and 3% humidified O_2–N_2 (21:79 vol%) as oxidant under OCV condition for (a) Cell-97 and (b) Cell-99.

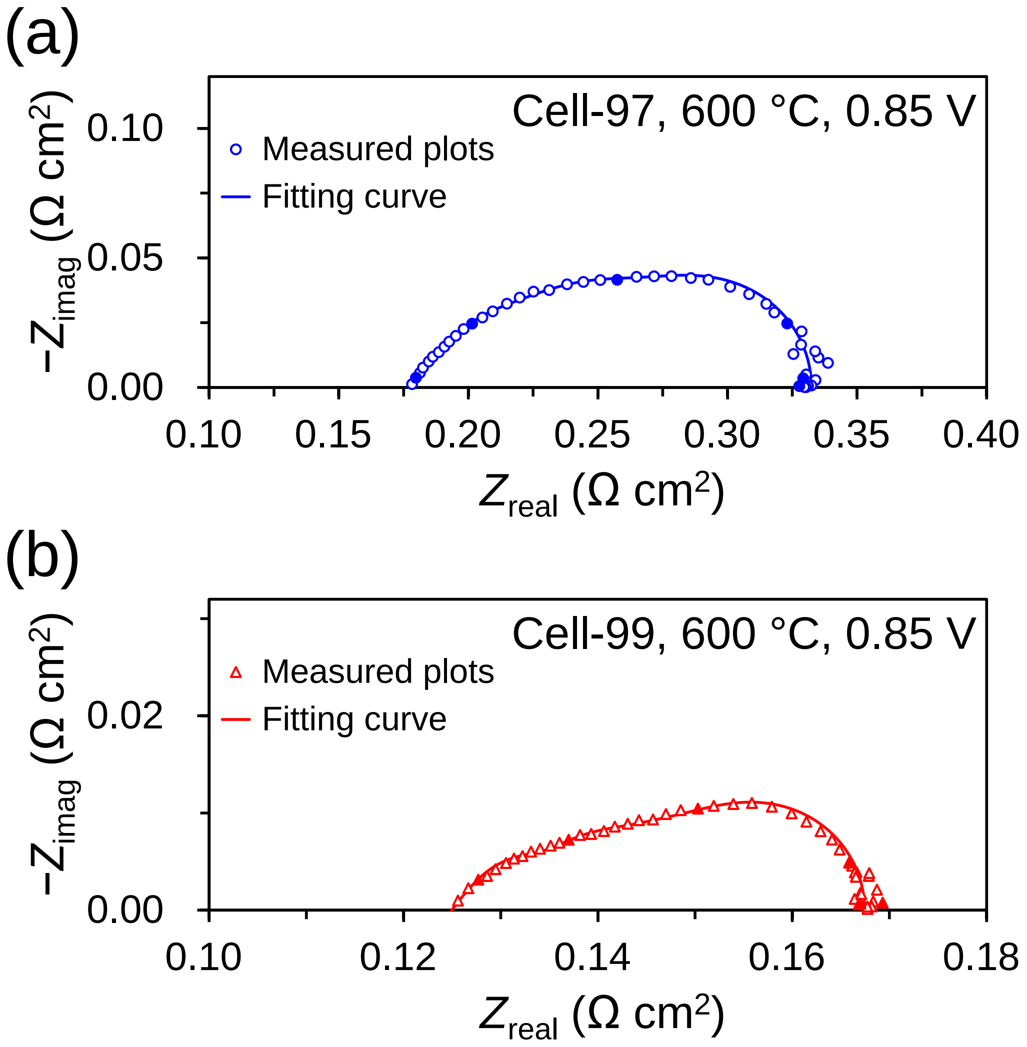

Figure S9

Fitting curves for impedance spectra at 600 °C using 3% humidified H_2 as fuel and 3% humidified O_2–N_2 (21:79 vol%) as oxidant under operating condition of 0.85 V for (a) Cell-97 and (b) Cell-99.

Table S1

Cation concentration of Ba, Zr, Yb, and Ni of BZYb20 electrolyte surface of Cell-97 and Cell-99 determined by XRF.

Sample	Cation concentration (at%)				A/B ratio
	Ba	Zr	Yb	Ni	
Cell-97	48.7	45.1	4.8	1.3	0.95
Cell-99	48.9	45.5	4.7	0.9	0.96

Table S2

Detailed maximum power densities (MPDs) and open-circuit voltages (OCVs) of Cell-97 and Cell-99.

Temperature	Cell-97		Cell-99	
(°C)	MPD ($W\ cm^{-2}$)	OCV (V)	MPD ($W\ cm^{-2}$)	OCV (V)
700	1.196	0.915	1.704	0.880
650	1.000	0.962	1.555	0.933
600	0.787	0.995	1.306	0.980
550	0.589	1.019	1.013	1.020
500	0.411	1.039	0.725	1.052

Table S3

Detailed components of previously reported SOFCs and PCFCs shown in Fig. 5. Note that the thicknesses of electrolyte are approximate values.

Components			Ref. In Fig. 5	Ref.
Cathode	Electrolyte	Anode		
LBC-BZYb10	BZYb20 (~5 μm)	Ni-BZYb20	This work (Cell-99)	
LSCF-GDC	GDC (~1 μm) / ScSZ (~5 μm)	Ni-ScSZ	46	1
STFC:PrO_x	GDC (~1 μm) / YSZ (~1.5 μm)	Ni-YSZ	47	2
SSC-SDC	GDC (~3 μm) / YSZ (~3 μm)	Ni-YSZ	48	3
BCFZY-BCZY63	BZCYYb1711 (10–20 μm μm)	Ni-BZCYYb1711	49	4
PBSCF	BZCYYb4411 (~15 μm)	Ni-BZCYYb4411	50	5
BSCF	BCZY3 (~5 μm)	Ni-BCZY3	51	6
BCCY	BZCYYb1711 (~16 μm)	Ni-BZCYYb1711	52	7
Catalyst-coated LSCF	BZCYYb1711 (~10 μm)	Ni-BZCYYb1711	53	8
LSC	BZY15 (~2.5 μm)	Ni-BZY15	54	9
BCFZY	BZY20 (10–20 μm)	Ni-BZY20	55	10
PBC-impregnated BZY20	BZY20 (~15 μm)	Ni-BZY20	56	11

LSCF: $La_{0.6}Sr_{0.4}Co_{0.2}Fe_{0.8}O_{3-\delta}$
GDC: Gd-doped CeO_2
ScSZ: Sc_2O_3-stabilized ZrO_2
STFC: $Sr(Ti_{0.3}Fe_{0.63}Co_{0.07}) O_{3-\delta}$
YSZ: Y_2O_3-stabilized ZrO_2
SSC: $Sm_{0.5}Sr_{0.5}CoO_{3-\delta}$
SDC: Sm-doped CeO_2
BCFZY: $BaCo_{0.4}Fe_{0.4}Zr_{0.1}Y_{0.1}O_{3-\delta}$
BCZY63: $BaCe_{0.6}Zr_{0.3}Y_{0.1}O_{3-\delta}$
BZCYYb1711: $BaZr_{0.1}Ce_{0.7}Y_{0.1}Yb_{0.1}O_{3-\delta}$
PBSCF: $PrBa_{0.5}Sr_{0.5}Co_{1.5}Fe_{0.5}O_{5+\delta}$
BZCYYb4411: $BaZr_{0.4}Ce_{0.4}Y_{0.1}Yb_{0.1}O_{3-\delta}$
BSCF: $Ba_{0.5}Sr_{0.5}Co_{0.8}Fe_{0.2}O_{3-\delta}$
BCZY3: $BaCe_{0.55}Zr_{0.3}Y_{0.15} O_{3-\delta}$
BCCY: $BaCo_{0.7}(Ce_{0.8}Y_{0.2})_{0.3}O_{3-\delta}$
LSC: $La_{0.6}Sr_{0.4}Co O_{3-\delta}$
BZY15: $BaZr_{0.85}Y_{0.15}O_{3-\delta}$
BZY20: $BaZr_{0.8}Y_{0.2}O_{3-\delta}$
PBC: $PrBaCo_2O_{5+\delta}$

Table S4

Summary of CNLS fitting results with equivalent circuit model for Cell-97 and Cell-99 at 600 °C using 3% humidified H_2 as fuel and 3% humidified O_2–N_2 (21:79 vol%) as oxidant under operating condition of 0.85 V.

Sample	Equivalent circuit model parameters										
	R_0 (Ω cm^2)	R_1 (Ω cm^2)	C_1 (F cm^{-2})	R_2 (Ω cm^2)	C_2 (F cm^{-2})	R_3 (Ω cm^2)	C_3 (F cm^{-2})	R_4 (Ω cm^2)	C_4 (F cm^{-2})	R_5 (Ω cm^2)	C_5 (F cm^{-2})
Cell-97	0.1644	0.0136	4.55×10^{-6}	0.0122	3.05×10^{-5}	0.0308	2.22×10^{-4}	0.0464	1.03×10^{-3}	0.0648	5.80×10^{-3}
Cell-99	0.1160	0.0107	4.59×10^{-6}	0.0080	3.45×10^{-5}	0.0086	2.60×10^{-4}	0.0063	0.95×10^{-3}	0.0178	4.16×10^{-3}

References

[1] Sato, K.; Iwata, C.; Kannari, N.; Abe, H. Highly Accelerated Oxygen Reduction Reaction Kinetics in Colloidal Processing-Derived Nanostructured Lanthanum Strontium Cobalt Ferrite/Gadolinium-Doped Ceria Composite Cathode for Intermediate-Temperature Solid Oxide Fuel Cells. *J. Power Sources* **2019**, *414*, 502–508.

[2] Park, B.-K.; Scipioni, R.; Zhang, Q.; Cox, D.; Voorhees, P. W.; Barnett, S. A.; Tuning Electrochemical and Transport Processes to Achieve Extreme Performance and Efficiency in Solid Oxide Cells. *J. Mater. Chem. A* **2020**, *8*, 11687–11694.

[3] Shimada, H.; Sumi, H.; Yamaguchi, Y.; Nomura, K.; Mizutani, Y.; Fujishiro, Y.; Shin, W. *J. Power Sources* **2023**, *563*, 232781.

[4] Duan, C.; Tong, J.; Shang, M.; Nikodemski, S.; Sanders, M.; Ricote, S.; Almansoori, A.: O'Hayre, R. Readily Processed Protonic Ceramic Fuel Cells with High Performance at Low Temperatures. *Science* **2015**, *349*, 1321–1326.

[5] Choi, S.; Kucharczyk, C. J.; Liang, Y.; Zhang, X.; Takeuchi, I.; Ji, H.-I.; Haile, S. M. Exceptional Power Density and Stability at Intermediate Temperatures in Protonic Ceramic Fuel Cells. *Nat. Energy* **2018**, *3*, 202–210.

[6] An, H.; Lee, H.-W.; Kim, B.-K.; Son, J.-W.; Yoon, K. J.; Kim, H.; Shin, D.; Ji, H.-I.; Lee, J.- H. A 5 × 5 cm^2 Protonic Ceramic Fuel Cell with a Power Density of 1.3 W cm^{-2} at 600 °C. *Nat. Energy* **2018**, *3*, 870–865.

[7] Song, Y.; Chen, Y.; Wang, W.; Zhou, C.; Zhong, Y.; Yang, G.; Zhou, W.; Liu, M.; Shao, Z. Self-Assembled Triple-Conducting Nanocomposite as a Superior Protonic Ceramic Fuel Cell Cathode. *Joule* **2019**, *3*, 2842–2853.

[8] Niu, Y.; Zhou, Y.; Zhang, W.; Zhang, Y.; Evans, C.; Luo, Z.; Kane, N.; Ding, Y.; Chen, Y.; Guo, X.; Lv, W.; Liu, M. Highly Active and Durable Air Electrodes for Reversible

Protonic Ceramic Electrochemical Cells Enabled by an Efficient Bifunctional Catalyst. *Adv. Energy Mater.* **2022**, *12*, 2103783.

[9] Bae, K.; Jang, D. Y.; Choi, H. J.; Kim, D.; Hong, J.; Kim, B.-K.; Lee, J.-H.; Son, J.-W.; Shim, J. H. Demonstrating the Potential of Yttrium-Doped Barium Zirconate Electrolyte for High-Performance Fuel Cells. *Nat. Commun.* **2017**, *8*, 14553.

[10] Duan, C.; Kee, R. J.; Zhu, H.; Karakaya, C.; Chen, Y.; Ricote, S.; Jarry, A.; Crumlin, E. J.; Hook, D.; Braun, R.; Sullivan, N. P.; O'Hayre, R. Highly Durable, Coking and Sulfur Tolerant, Fuel-Flexible Protonic Ceramic Fuel Cells. *Nature* **2018**, *557*, 217–222.

[11] Bi, L.; Shafi, S. P.; Da'as, E. H.; Traversa, E. Tailoring the Cathode–Electrolyte Interface with Nanoparticles for Boosting the Solid Oxide Fuel Cell Performance of Chemically Stable Proton-Conducting Electrolytes. *Small* **2018**, *14*, 1801231.
